# The Role of Factor Inhibiting HIF (FIH-1) in Inhibiting HIF-1 Transcriptional Activity in Glioblastoma Multiforme

**DOI:** 10.1371/journal.pone.0086102

**Published:** 2014-01-23

**Authors:** Enfeng Wang, Chunyang Zhang, Navatha Polavaram, Fengming Liu, Gang Wu, Mark A. Schroeder, Julie S. Lau, Debabrata Mukhopadhyay, Shi-Wen Jiang, Brian Patrick O'Neill, Kaustubh Datta, Jinping Li

**Affiliations:** 1 Department of Biochemistry and Molecular Biology, Mayo Clinic Cancer Center, Rochester, Minnesota, United States of America; 2 Department of Neuro-Surgery, the First Affiliated Hospital of Baotou Medical College, Baotou, China; 3 Department of Biochemistry and Molecular Biology and Eppley Cancer Center, University of Nebraska Medical Center, Omaha, Nebraska, United States of America; 4 Department of Research and Development, Guangxi Medicinal Botanical Institute, Nanning, Guangxi, China; 5 Department of Radiation Oncology, Mayo Clinic Cancer Center, Rochester, Minnesota, United States of America; 6 Department of Biomedical Science, Mercer University School of Medicine, Savannah, Georgia, United States of America; 7 Department of Obstetrics and Gynecology, Memorial Health Hospital, Savannah, Georgia, United States of America; Ohio State University, United States of America

## Abstract

Glioblastoma multiforme (GBM) accounts for about 38% of primary brain tumors in the United States. GBM is characterized by extensive angiogenesis induced by vascular growth factors and cytokines. The transcription of these growth factors and cytokines is regulated by the Hypoxia-Inducible-Factor-1(HIF-1), which is a key regulator mediating the cellular response to hypoxia. It is known that Factor Inhibiting HIF-1, or FIH-1, is also involved in the cellular response to hypoxia and has the capability to physically interact with HIF-1 and block its transcriptional activity under normoxic conditions. Delineation of the regulatory role of FIH-1 will help us to better understand the molecular mechanism responsible for tumor growth and progression and may lead to the design of new therapies targeting cellular pathways in response to hypoxia. Previous studies have shown that the chromosomal region of 10q24 containing the FIH-1 gene is often deleted in GBM, suggesting a role for the FIH-1 in GBM tumorigenesis and progression. In the current study, we found that FIH-1 is able to inhibit HIF-mediated transcription of GLUT1 and VEGF-A, even under hypoxic conditions in human glioblastoma cells. FIH-1 has been found to be more potent in inhibiting HIF function than PTEN. This observation points to the possibility that deletion of 10q23-24 and loss or decreased expression of FIH-1 gene may lead to a constitutive activation of HIF-1 activity, an alteration of HIF-1 targets such as GLUT-1 and VEGF-A, and may contribute to the survival of cancer cells in hypoxia and the development of hypervascularization observed in GBM. Therefore FIH-1 can be potential therapeutic target for the treatment of GBM patients with poor prognosis.

## Introduction

The transcription factor Hypoxia Inducible Factor-1/2(HIF-1/2α) regulates and coordinates the expression of an extensive array of genes required to maintain oxygen homeostasis in every living system during conditions of severe oxygen stress [1]–[3]. Inhibiting HIF-1α function in the tumor microenvironment is important for suppressing tumor angiogenesis and increasing tumor cell apoptosis [4]. Of particular importance, a HIF-1α inhibitor, 2-methoxyestradiol, showed a dose-dependent decrease in tumor growth and angiogenesis in a rat orthotopic brain tumor model, underscoring the importance of inhibiting HIF in Glioblastoma multiforme (GBM) [5]. GBM is a highly proliferative and infiltrating primary brain tumor that contains regions of hypervascularization (angiogenesis) [6]–[8]. Because surgery, radiation, and chemotherapy have been unsuccessful in treating malignant gliomas [9]–[11], it is critically important to develop new therapeutic regimens for use along with the established treatment options. Structurally, HIF-1 is a heterodimer of two basic helix-loop-helix PAS domain proteins, namely HIF-1α and HIF-1β [12], [13]. The HIF-1β subunit is constitutively detected in the nucleus, whereas the protein stability of the HIF-1α subunit and its isoforms (HIF-2α and HIF-3α) depends upon the cellular oxygen concentration [12], [14], [15]. In normoxic condition, the ubiquitin-proteasome pathway rapidly degrades HIF-αsubunits whose steady state levels are low, and as a result the transcriptionally active complex cannot form [16]–[20]. Detailed molecular studies demonstrated that in high oxygen concentrations, HIF-1α is hydroxylated at the proline residue of its oxygen-dependent degradation domain [21]–[23].This post-translational modification of HIF-1α is necessary for degradation by the proteasome complex [17], [24].

Stabilization of HIF-1α protein levels is not sufficient for the transcriptional activation of its target genes. In oxygenated cells, HIF-1α is also hydroxylated at an asparagine residue at the COOH-terminal transactivation domain (CAD). As a result, the HIF complex cannot bind to the adaptor protein p300 to execute its transcriptional activity under normoxic conditions [25]–[30]. In this regard, the status of Factor Inhibiting HIF-1 (FIH-1) may be important in HIF-mediated gene transcription in glioblastoma. FIH-1, presently identified as an asparagine hydroxylase [29], [31], [32], specifically hydroxylates the transcription factor Hypoxia Inducible Factor-1/2α (HIF-1/2α under normal physiological conditions and inhibits its association with the adaptor protein p300 [28], [33], [34]. FIH-1 protein is widely expressed in human tissues and is thus potentially available for the regulation of HIF activity across a broad range of cells and culture conditions [35]. It is also suggested that FIH-1 has important non-redundant effects *in vivo* on the expression of a range of HIF transcriptional targets in normoxia and even in hypoxia [35]. Importantly, *FIH-1* is located at chromosome 10q24, which is often deleted in glioblastoma, especially in secondary glioblastoma, making it an important candidate gene to study the progression of this cancer [36]–[41], while mutation of *PTEN* at 10q23.3 are common (12–40%) in primary glioblastoma [40], [42]–[44]. Due to these reported details regarding FIH-1, we seek to determine the importance of the FIH-1 gene in GBM progression. Our search in ONCOMINE database also showed a significant reduction of FIH-1 mRNA levels in GBM compared to normal brain tissues (figure S1) [45]. This prompted us to further examine if the loss or decreased expression of FIH-1 played any role in HIF-mediated gene expression in GBM. We also compared the function of FIH-1 with that of PTEN in the synthesis of HIF-regulated genes including GLUT-1 and VEGF-A. Our studies clearly emphasize the importance of FIH-1 in regulating HIF-mediated genes in a subset of primary glioblastoma cases without *PTEN* mutation.

## Materials and Methods

### Cell Culture

The human glioblastoma cell line U87 MG (ATCC # HTB-14) and the serially transplanted GBM cell lines (developed at the Mayo Clinic) [46] were cultured at 37°C in RPMI 1640 with L-glutamine (Mediatech) supplemented with penicillin/streptomycin and 10% normal fetal bovine serum (FBS) (Hyclone Laboratories). To induce hypoxia (3% O_2_), the cells need to be placed in hypoxia chamber (Heracells 150i tris-Gas Incubators with O_2_ control from 1% to 21%, Thermo Scientific, Saheville, NC) which is preset to 3% of O_2_ for at least 4 hrs and maintains a sub-ambient O_2_ level for 12–48 hrs by the regulated injection of N_2_. The CO_2_ and moisture content remains same as regular tissue culture incubator as previously reported [47]–[49].

### Nuclear Extract Preparation

The glioblastoma cell suspensions were incubated in a hypotonic buffer [10 mM HEPES (pH 7.8), 10 mM KCl, 2 mM MgCl_2_, 0.1 mM EDTA, 10 µg/ml aprotinin, 3 mM dithiothreitol, and 0.2 mM phenylmethylsulfonylfloride (PMSF)] for 15 min on ice. The nonionic detergent IGEPAL (Sigma) (10%) was then added to the cell suspensions followed by vigorous mixing. After centrifugation at 14,000 rpm for 5 min, the pellets were resuspended in a hypertonic buffer solution: 50 mM HEPES (pH 7.8), 50 mM KCl, 300 mM NaCl, 0.1 mM EDTA, 10 µg/ml aprotinin, 3 mM dithiothreitol, and 0.2 mM phenylmethylsulfonylfloride (PMSF)] and mixed on a rotating rack for 25 min at 4°C. The samples were again centrifuged at 14,000 rpm for 10 min, and the supernatants were collected as nuclear extracts.

### Immunoprecipitation and Western Blot Analysis

Immunoprecipitation was performed with150 µg of nuclear protein extracts or whole cell extracts using antibodies (1 µg) directed against HIF-1α or p300 and then pulled down with Protein A-agarose beads (Pharmacia, New York, NY). For Western blots, antibodies against HIF-1α (Novus Biologicals, Littleton, CO) and p300 (Santa Cruz Biotechnology, Santa Cruz, CA), FIH-1 (Novus Biologicals), and PTEN (Cell Signaling Technology, Denver, MA) were used.

### FACS analysis

U87 cells were washed twice with PBS and incubated with 4 ml of collagenase solution (0.2 µg/ml collagenase, 0.2 µg/ml soybean trypsin inhibitor, 1 µg/ml BSA, and 2 mM EDTA in PBS) at 37°C for 30 min. Cells were then detached with gentle scraping and collected by centrifugation at 1100 rpm for 3 min. Cell pellets were washed twice with cold PBS containing 0.1% BSA and resuspended in the same buffer at 0.5×10^6^ cells/ml. 200 µl of the cell suspension containing 1 µg of mouse anti-GLUT-1 antibody (R&D Systems, Minneapolis, MN) or mouse IgG and incubated at 4°C for 1 hour. Cells were centrifuged at 1300 rpm for 3 min. Cell pellets were again washed twice and resuspended in 100 µl of the PBS solution containing 2.5 µg/ml fluorescein isothiocyanate-conjugated anti-mouse IgG antibody. After incubation at 4°C for 30 min, cells were washed twice and resuspended in 400 µl of the PBS solution. FACS analysis was carried out using a FACSCalibur flow cytometer (BD Biosciences) and the CellQuest software.

### RNA Isolation and Real-Time Polymerase Chain Reaction

After washing twice with ice cold PBS, total RNA was isolated from U87 MG and GBM lines using the RNeasy Mini kit (Qiagen). mRNA levels of GLUT-1, VEGF-A, FIH-1 were measured with Taqman real-time PCR method. The sequence for forward, reverse, and Taqman middle primers for human GLUT-1, VEGF-A, FIH-1 and for human 36B4 (housekeeping gene) were taken from the PubMed gene bank and synthesized (Integrated DNA Technologies).

GLUT-1 forward: 5′-GCG GAA TTC AAT GCT GAT GAT-3′. GLUT-1 reverse: 5′-CAG TTT CGA GAA GCC CAT GAG-3′. GLUT-1 middle primer: 5′-CT GGC CTT CGT GTC CGC CGT-3′.

VEGF-A forward: 5′-TAC CTC CAC CAT GCC AAG TG-3′. VEGF-A reverse: 5′-GAT GAT TCT GCC CTC CTC CTT-3′. VEGF-A middle primer: 5′-TCC CAG GCT GCA CCC ATG GC -3′. FIH-1 forward primer: 5′-GCC AGC ACC CAC AAG TTC TT-3′.FIH-1 reverse primer: 5′-CCT GTT GGA CCT CGG CTT AA-3′. FIH-1 middle primer: 5′-GTT CTG GAA ATT GGC CAT CTT CTT CTC ATC A-3′. 36B4 forward: 5′-ATG CAG CAG ATC CGC ATG T-3′. 36B4 reverse: 5′-TCA TGG TGT TCT TGC CCA TCA-3′. 36B4 middle primer: 5′-CAC CAC AGC CTT CCC GCG AA-3′. Each real-time PCR reaction was performed using 1.0 µg total RNA, 12.5 µl RT-PCR Master Mix (Applied Biosystems), 0.6 µl RNase inhibitor (Applied Biosystems), 50 nM forward primer, 50 nM reverse primer, and 100 nM middle primer. Reverse transcription was performed at 48°C for 30 min, followed by a 10 min incubation at 95°C to inactivate the reverse transcriptase. For the real-time PCR step, forty cycles of 95°C for 15 sec and 60°C for 1 min were performed with an ABI Prism 7700 Sequence Detector (Applied Biosystems). All experiments were performed three times, and, from each of the three, triplicate readings were taken and the average was calculated. The relative RNA amount was calculated as follows: Δ = CT (GLUT-1/VEGF-A sample)-CT (36B4 sample). ΔΔ = Δ (transfected sample)-Δ (empty vector sample). Relative RNA amount in comparison to the control = 2^−ΔΔ^. The average and standard deviation from three experiments were also calculated. p value was calculated with *t*-test, * represents p value is less than 0.05.

### Immunohistochemistry staining

Tissue sections were prepared by Drs. C. David James and Jann Sarkaria's group at Mayo Clinic [46], [50]. An immunohistochemistry accessory kit (Bethyl laboratories, Inc., Montgomery, TX) was used to examine expression levels of FIH-1 according to the manufacturer's protocol. Briefly, after deparaffinization and rehydration, the slides were incubated in 3% hydrogen peroxide/methanol to quench endogenous peroxidase. Slides were then incubated in hot Epitope retrieval buffer (provided in kit) to recover epitopes for 20 min at 90–96°C. Non-specific reactions were blocked by incubating the sections with blocking reagent (included in the kit) for 30 min at room temperature, then 200 µl of human FIH-1 antibody (dilution 1∶200,catalog ab92498, Abcam, Cambridge, MA) was applied to each slide and incubated overnight at 4°C. Slides were then incubated with working anti-rabbit IHC antibody (provided in kit), and peroxidase activity was visualized with working 3, 3′-diaminobenzidine (DAB) substrate (provided in kit). Counterstaining was performed with hematoxylin. The stained sections were randomly selected high-power fields (×40).

## Results

### FIH-1 decreases GLUT-1 mRNA levels and thus protein levels in U87 under normoxia and hypoxia

**Figure 1:**
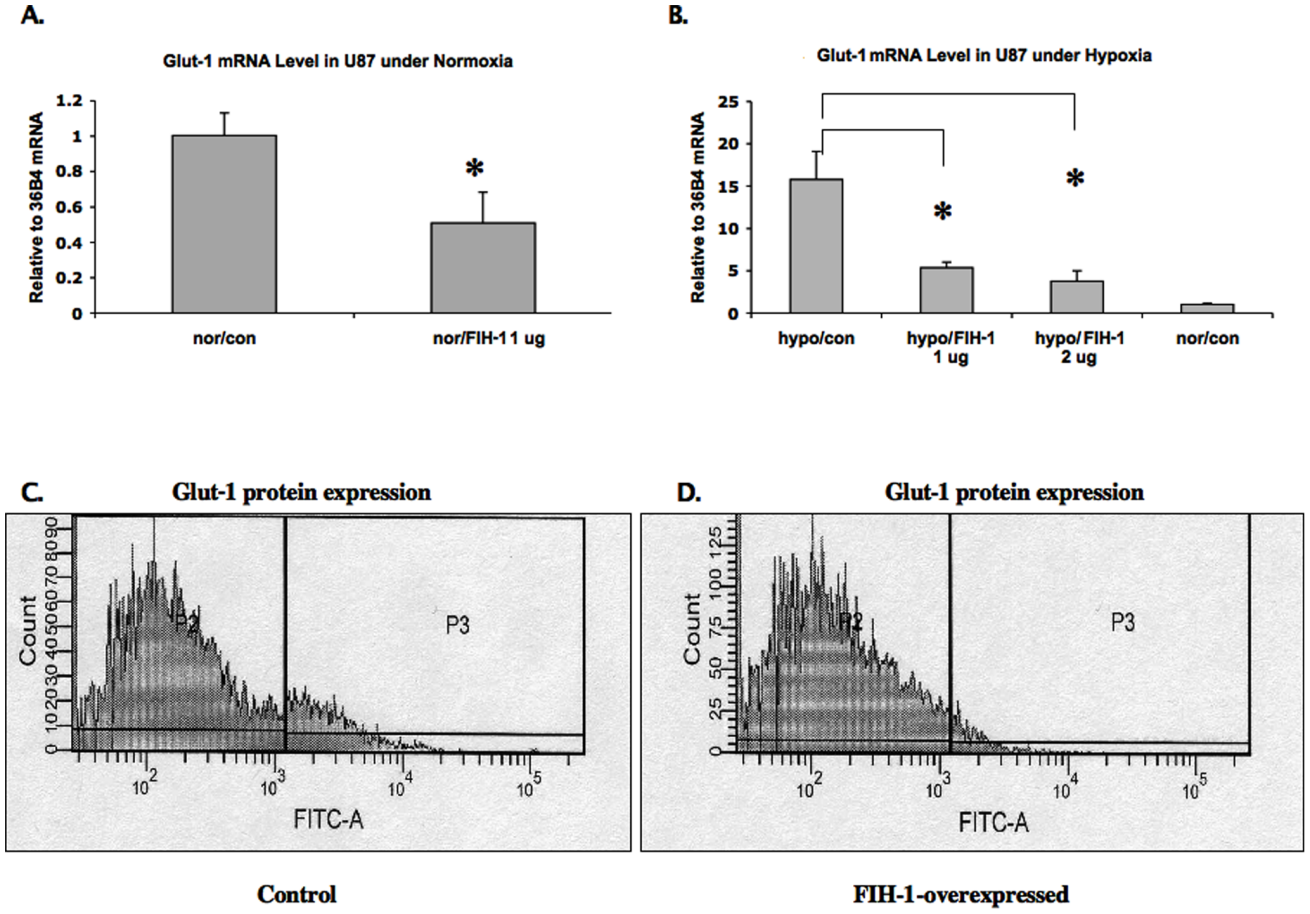
The mRNA levels of GLUT-1 were reduced with FIH-1 overexpression. 1A and 1B: U87 cells were transfected with FIH-1-expression plasmid and cultured in both hypoxic (1B) and normoxic (1A) conditions. Total RNA was harvested and RQ-PCR was performed using primers specific for GLUT-1 and 36B4 (internal control). A significant decrease of GLUT-1 mRNA levels was observed in cells transfected with FIH-1 expression plasmid in both normoxia and hypoxia. The data here is the mean from three independent results. * Represents p value less than 0.05. 1C and 1D: The expression levels of GLUT-1 were decreased by overexpression of FIH-1 in U87 cells. U87 cells were transfected with constructs either expressing FIH-1 or with vector alone and incubated for 48 hrs under normoxic condition. Cells were collected and subjected to flow cytometry analysis. A reduction in GLUT-1 levels was detected in cells transfected with FIH-1 plasmid (1D) compared with cells transfected with control vector (1C), as shown in the P3 population.

U87 cell line, were chosen for its tolerance to the transient transfection manipulation in this study although it expresses minimal basal levels of FIH-1. U87 cells were transfected with plasmid expressing FIH-1 under normaxic and hypoxic conditions, and expression of GLUT-1 was examined. First, we observed a decrease in the level of GLUT-1 mRNA, the HIF-1α target gene [3], [6], in U87 cells after overexpressing FIH-1 (Figure 1A). Interestingly, we also observed a decrease in the level of GLUT-1 mRNA even under hypoxic conditions, demonstrating that FIH-1 regulates HIF activity in U87 cells even in hypoxia (Figure 1B), which suggests that hypoxia is not the sole factor for inducing HIF-regulated genes in GBM [6] and FIH-1 involved (mediated) HIF activity regulation may also play role during GBM tumor progression in certain advanced cases. This decrease in GLUT-1 mRNA levels corresponds with decreased GLUT-1 protein levels, as observed by FACS analysis (Figure 1C and 1D).

### PTEN cannot inhibit GLUT-1 transcription in U87 under hypoxia

**Figure 2:**
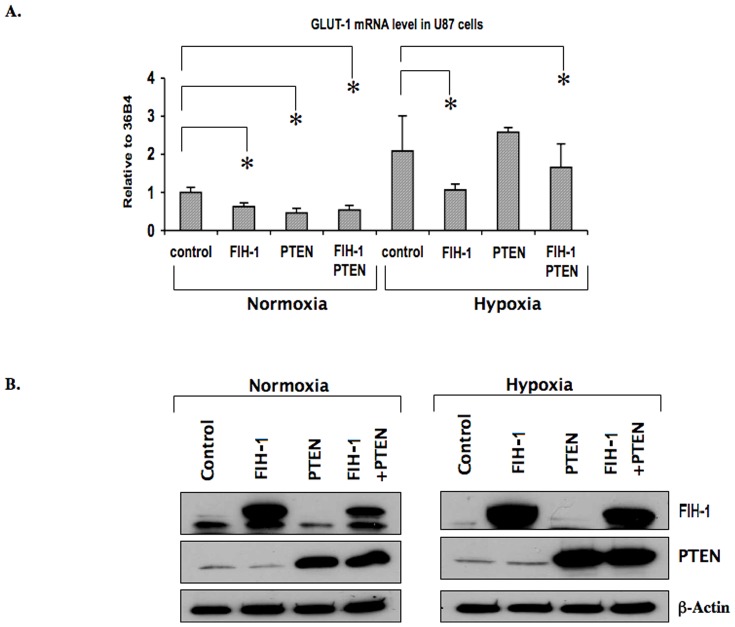
Inhibit GLUT-1 transcription by FIH-I in U87 under hypoxia GLUT-1 expression was inhibited by PTEN under normoxic condition. U87 cells were transfected with FIH-1 and PTEN expression plasmids either alone or in combination and cultured in both hypoxic and normoxic conditions. 2A: Total RNA was obtained and followed with RQ-PCR using primers specific for GLUT-1 and 36B4 (internal control). A significant reduction of GLUT-1 mRNA levels was observed in cells expressing PTEN, FIH-1, or both under normoxic conditions, but interestingly, GLUT-1 mRNA levels were not changed in cells expressing PTEN under hypoxic conditions. Average and standard deviation of three independent experiments were calculated. * Represents p value less than 0.05. 2B: Whole cell lysates from each experimental condition were collected and protein levels of FIH-1 (top panel) and PTEN (middle panel) were examined by Western blot. β-actin was used as a loading control (lower panel).

PTEN has been shown to be functionally inactive in many primary GBM cell lines [40], [42]–[44], [51], [52]. Several reports have suggested that the antiangiogenic function of PTEN is due to its inhibitory role within the PI3K-AKT signaling pathway [53]. Because this pathway has been found to be important for HIF-1α synthesis in various cancers [54], we wanted to study the effect of PTEN in combination with FIH-1 on HIF activity in GBM cell line-U87-MG. We observed a decrease in the level of GLUT-1 mRNA in U87 cells when overexpressing PTEN under normoxic conditions (Figure 2A). This decrease in GLUT-1 mRNA was almost comparable to that of U87 cells overexpressing FIH-1. However, we did not observe any synergistic effects on the level of GLUT-1 mRNA when both FIH-1 and PTEN were overexpressed under normoxic conditions. Interestingly, in hypoxia, FIH-1 overexpression in U87 showed a significant decrease in GLUT-1 mRNA, but the overexpression of PTEN alone was not sufficient to decrease GLUT-1 mRNA (Figure 2A). This result therefore suggests a more potent FIH-1 function as compared to PTEN in regulating HIF activity in glioblastoma, especially under hypoxic conditions. Figure 2B shows the amount of FIH-1 and PTEN proteins in U87 cells after transfection with the FIH-1 and PTEN overexpressing plasmids.

**Figure 3:**
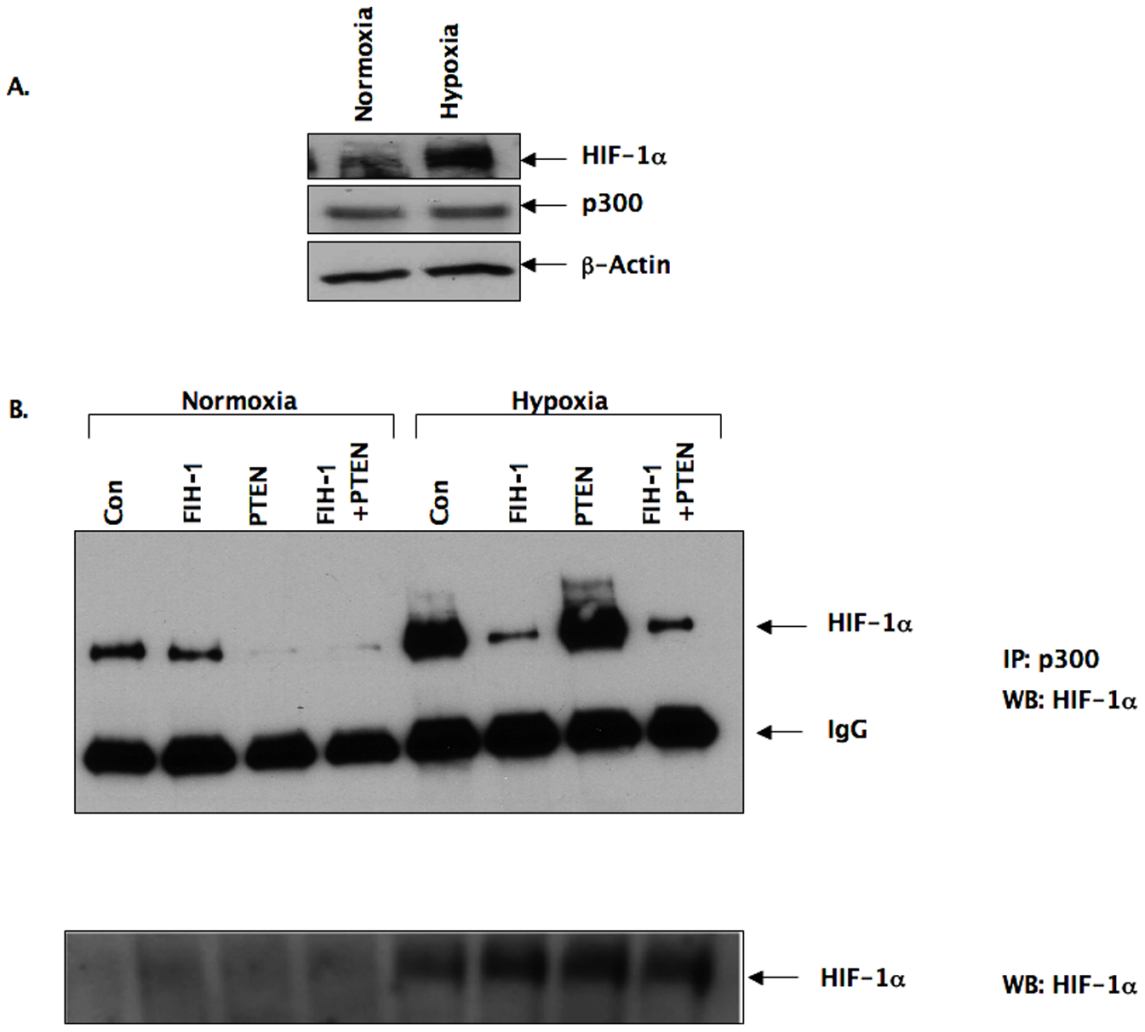
Association of HIF-1α with p300 in the presence of FIH-1, PTEN in U87 cells. A: Western blot of HIF-1α from protein lysates of U87 cells incubated overnight either in hypoxic (3% oxygen) or normoxic (21% oxygen) conditions (top panel). No change of p300 protein levels was observed in the same samples (middle panel). β-actin was used as a loading control (lower panel). B: Association of HIF-1α with p300 in U87 cells: cells were transiently transfected with either FIH-1 or a PTEN expression plasmid, or both of them together for 24 hrs, and then incubated overnight under both normoxic and hypoxic conditions. Nuclear extracts were collected and subjected to immunoprecipitation with anti-p300 antibody followed by Western blot analysis with anti-HIF-1α (top panel) and IgG (middle panel). Nuclear extracts were also subjected to western blot analysis with anti- HIF-1α antibody (bottom panel).

In order to provide a mechanistic explanation for the result described in Figure 2A, we studied the association of HIF-1α with p300 in U87 cells in the presence of FIH-1, PTEN, or both under normoxic as well as hypoxic conditions (Figure 3B). The association of HIF-1α with p300 is essential for HIF-1 transcriptional activity. We observed a detectable amount of HIF-1α protein in U87 under normoxia (Figure 3A and 3B) and also a clear association between HIF-1α and p300 under the same conditions (Figure 3B). This suggests that residual HIF-1 activity exists in this cancer cell line even under normoxic conditions. As in hypoxia, the U87 cells expressed high levels of HIF-1α (Figure 3B, bottom panel), and we also observed an increase in the association of HIF-1α with p300 (Figure 3B), which confirms the high HIF-1 activity under these conditions (Figure 2A). Interestingly, overexpression of FIH-1, but not PTEN, inhibited HIF-1 activity by preventing the association of p300 with HIF-1α Figure 3B). The amount of p300 immunoprecipitated was same in each condition as shown in the middle panel of Figure 3B. This result, therefore, provides an explanation for the decrease in GLUT-1 levels under hypoxia in the presence of overexpressed FIH-1 alone (Figure 2A). On the other-hand, PTEN appears to regulate HIF activity only under normoxia.

### FIH-1 decreases VEGF-A mRNA in U87

**Figure 4:**
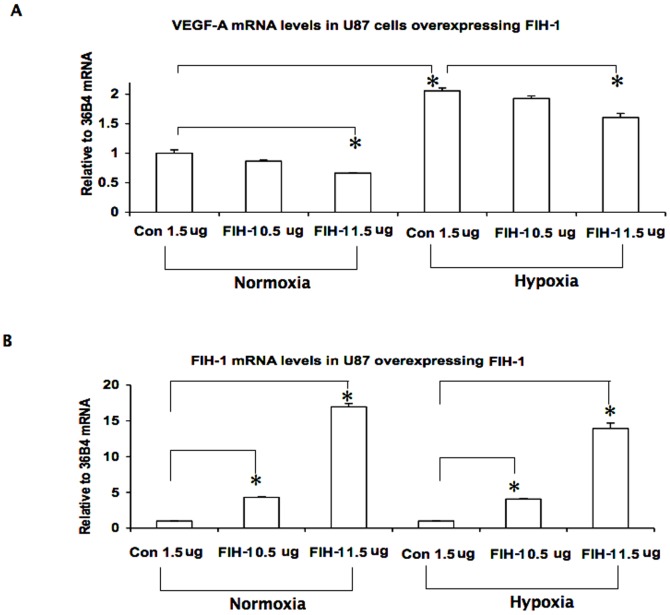
Inhibition of VEGF-A expression by FIH-1. VEGF-A expression was controlled by FIH-1 in both normoxia and hypoxia. U87 cells were transfected with the FIH-1 expression plasmid at different doses (e.g. 0.5 µg/ml, 1.5 µg/ml) and cultured under both hypoxic and normoxic conditions. Total RNA was obtained and then RQ-PCR was performed using primers specific for VEGF-A and 36B4 (internal control). VEGF-A mRNA levels were decreased with FIH-1 overexpresssion under both normoxic and hypoxic conditions (Figure 4A). FIH-1 mRNA levels were also measured as a control experiment (Figure 4B). The data here represent the mean of three independent experiments, and * represents p<0.05.

Apart from GLUT-1, we also examined FIH-1 regulation on vascular endothelial growth factor-A (VEGF-A) expression, another HIF target gene [7], [55]. Like GLUT-1, overexpression of FIH-1 in U87 cells down-regulated VEGF-A mRNA levels under normoxic conditions and, importantly, also in hypoxia (Figure 4). Interestingly, the expression levels of GLUT-1 and VEGF-A are different under the HIF-FIH regulation. Glut-1 transcription appears to be more sensitive than VEGF-A. The different responses to HIF may be due to the constitutive structural divergence in their promoter regions harboring the hypoxia response elements.

### Inverse correlation of the expression pattern of FIH-1 and VEGF-A in serially transplantable GBM cell lines

**Figure 5:**
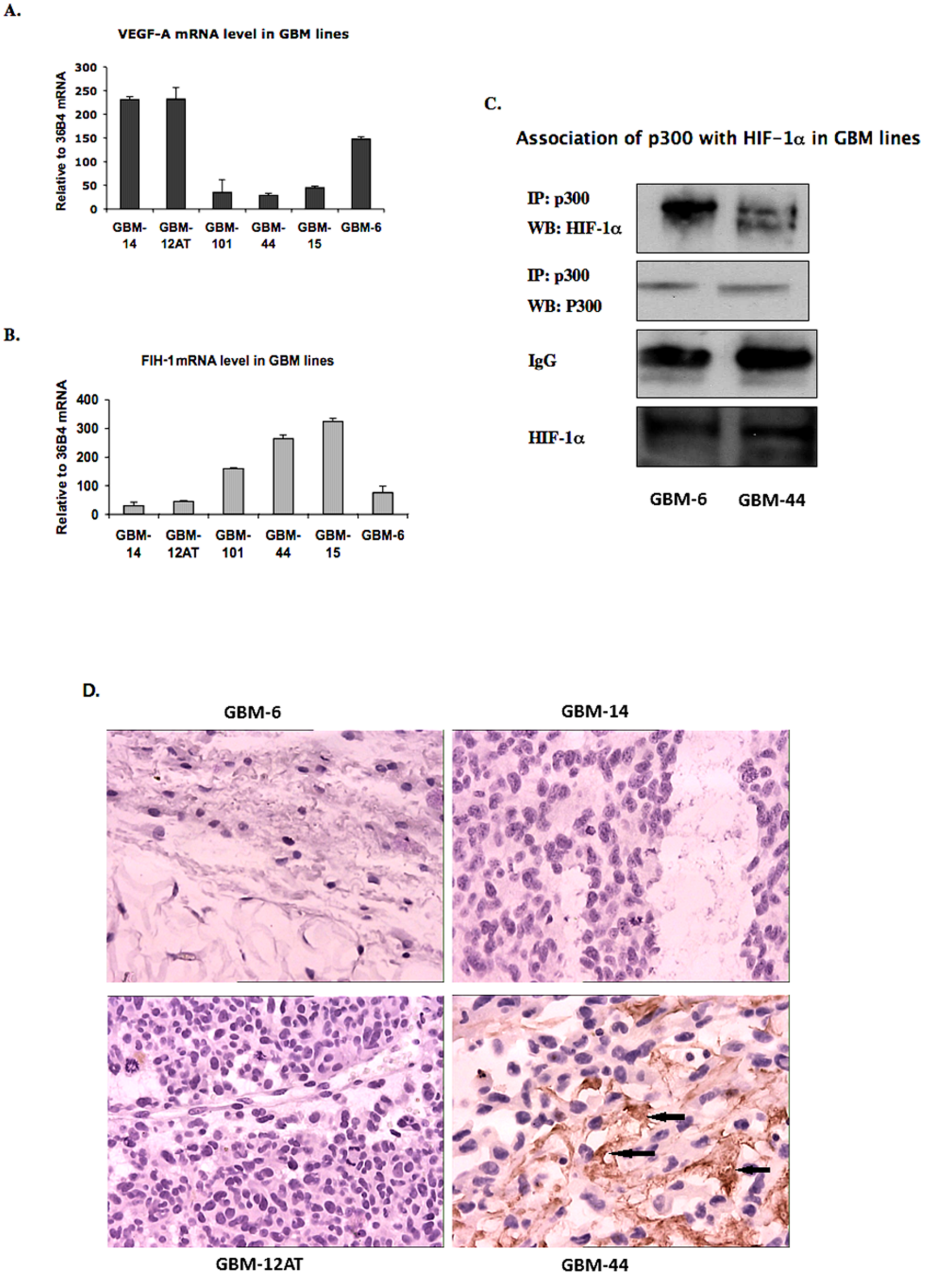
The expression pattern of FIH-1 and VEGF-A in serially transplantable GBM cell lines. 5A: The levels of VEGF-A mRNA were determined in different transplantable GBM xenografts by RQ-PCR. All values were normalized to the housekeeping gene 36B4. 5B: The levels of FIH-1 mRNA were determined in different transplantable GBM xenografts by real-time PCR. Values were normalized to the housekeeping gene 36B4. The levels of VEGF-A mRNA is inversely correlated with the levels of FIH-1 mRNA (correlation coefficient value r = −0.869, p = 0.025 (2-tailed), Pearson regression analysis was performed using SPSS software version 19.0). 5C: The association of p300 and HIF-1α was confirmed in GBM mouse xenograft cell line 6 and 44. The amount of p300 protein immunoprecipitated was same for each line. They also expressed similar levels of HIF-1α (bottom panel). 5D: FIH-1 expression in GBM xenografts by Immunohistostaining. High expression of FIH-1 was detected in GBM-44 line (brown color, marked as arrows), whereas no detectable levels of FIH-1 were found GBM-6, -12AT and -14.

In order to understand the significance of the loss of FIH-1 in GBM, we studied the expression levels of FIH-1 and VEGF-A in GBM lines other than U87 as well. We chose a panel of serially transplantable GBM xenografts that were initially established by direct implantation of patient GBM specimens into the flanks of nude mice [46]. These xenograft lines also grow as intracranial xenografts and retain the histopathologic features of their derivative GBM tumors. We examined the relative mRNA levels of VEGF-A and FIH-1 in these GBM xenograft lines, and found that many of the lines expressed high levels of VEGF-A and low levels of FIH-1(Figures 5A and 5B). In contrast, for those lines with low levels of VEGF-A, elevated FIH-1 levels were detected, indicating an inverse correlation between VEGF-A and FIH-1 expression. Importantly, these contrasting level of VEGF-A and FIH-1 was also in accordance to the association of p300 and HIF-1α observed in the GBM mouse xenograft cell line 6 compared to line 44 (Figure 5C). Interestingly, both the GBM-6 and GBM-44 cell lines have wild type levels of PTEN [46] and expressed similar level of HIF-1α protein (Figure 5C, bottom panel). We further detected FIH-1 expression in GBM-44, GBM-6, -12AT and -14 lines by Immunohistochemistry using the paraffin slides derived from these lines, and there was a significant FIH-1 expression in GBM-44 line but FIH-1 was undetectable in GBM-6, -12AT and -14 lines (Figure 5D). In this respect, only the difference in FIH-1 levels is regulating HIF activity (Figure 5C) and VEGF-A expression (Figure 5A), further confirming the importance of FIH-1 in regulating HIF activity in GBM.

## Discussion

In this study, we addressed the consequence of the loss of FIH-1 function in glioblastoma cell lines. A portion of chromosome 10q23-24 is often deleted in glioblastoma, resulting in loss of the tumor suppressor gene PTEN in a subset of up to 40% in primary glioblastoma [40], [43], [44]. The transcriptional activity of HIF-1α is high in glioblastoma cells [1]. PTEN deletion leads to increased AKT activation and thus to enhanced synthesis of HIF-1α [54]. The *FIH-1* gene is located on the 10q arm and is close to PTEN. Therefore, loss of FIH-1 might also be a common occurrence in glioblastoma. “ONCOMINE” data also supports the same notion (figure S1). FIH-1 hydroxylates HIF and thereby inhibits its association with p300 [28], [56], which is a co-activator molecule required for HIF function. As such, loss of FIH-1 may account for increased HIF activity in glioblastoma. Therefore, we studied whether ectopically overexpressed FIH-1 could inhibit HIF-mediated gene transcription in the glioblastoma cell line U87 MG.

We compared the role of FIH-1 with that of PTEN in regulating HIF activity. We observed a decrease in GLUT-1 (a HIF-regulated gene) mRNA and protein levels after overexpressing FIH-1 (Figure 1). Importantly, FIH-1 could inhibit GLUT-1 levels even in hypoxia (Figure 2A), which underscores its ability to inhibit HIF-mediated gene transcription in glioblastoma in this condition. In this respect, we would like to point out that although it is a hydroxylase by nature and therefore requires oxygen as one of its substrates, FIH-1 unlike other hydroxylases, has the ability to function even at substantially low oxygen concentrations [35]. It is known that the prolyl hydroxylase, which is required for HIF's degradation, functions in a much higher oxygen concentration range as compared to FIH-1 [57]. Therefore, FIH-1 could still be in a functionally active state at a certain hypoxic stage that is sufficient for inactivating prolyl hydroxylase and stabilizing the HIF protein. Active FIH-1 in these conditions then interferes with HIF function. In this respect, the absence of FIH-1 activity may be a necessary step for hypoxia-induced HIF function in solid tumors in the advanced stage. On the other hand, we observed that PTEN could inhibit HIF function in normoxia but was unable to influence its activity in hypoxia (Figure 3B). As a result, the loss of FIH-1 seems to be more critical than the loss of PTEN in activating HIF to its full extent in hypoxia. Similar to GLUT-1, overexpression of FIH-1 in U87 cells decreased the levels of VEGF-A mRNA (another HIF mediated gene and important for tumor angiogenesis) in both normoxia and hypoxia (Figure 4) further confirming the importance of FIH-1 in regulating HIF activity in glioblastoma. It is noticed that GLUT-1 and VEGF-A showed different responses to HIF-FIH-1 regulation, which may be due to their intrinsic structural divergence of the hypoxia response element(s) and other cis-elements located in their promoter regions.

We expanded our study from the U87 cell line to a panel of serially transplantable GBM lines established at the Mayo Clinic [46], [50]. These GBM lines were initially established by direct implantation of primary patient specimens into the flanks of nude mice. These cell lines can recapitulate the features human glioblastoma better than U87 when they are implanted in mice, therefore gave us the opportunity to address the significance of FIH-1 in a more clinically relevant situation. Moreover, a single cell line study does not address the issues related to tumor heterogeneity and thus is not sufficient to determine the function of a gene in the progression of a solid tumor. We therefore used these serially transplantable GBM lines to determine the relative amounts of FIH-1 and VEGF-A. Our data suggests that the levels of VEGF-A mRNA in those GBM lines are inversely correlated with those of FIH-1 mRNA and therefore validated our results in U87 cell line. When we compared two GBM lines (GBM-6 and GBM-44) having wild-type levels of PTEN [46] but with different levels of FIH-1 (Figure 5D), This data is in parallel with mRNA expression data (Figure 5B) where we observed GBM-44 and -15 only expressed high FIH mRNA. GBM-6 and GBM-44 showed different levels of HIF activity, underscoring the significance of FIH-1 over PTEN in regulating HIF function in GBM (Figure 5A and 5B). Interestingly previous reports on these GBM lines also suggested that the low FIH-1 expressing lines showed rapid tumor progression and poor prognosis. Patients with GBM-6, -12AT and -14 die earlier than patients with GBM-44 and -15 [46]. Mice with orthotopic implantation with GBM-44 survived significantly more than mice with GBM-12AT and-14 lines [58]. These results therefore address the importance of FIH-1 in GBM development and progression, especially in the subset of primary glioblastomas with PTEN wild type.

In summary, this study suggested that loss of FIH-1 may be a crucial event in HIF mediated gene transcription in GBM and thereby important for its progression, but further in-depth studies involving human patient tissues to are needed to delineate the function of FIH-1 in the initiation and metastasis of GBM.

## Supporting Information

Figure S1 **Under-expression of FIH-1 (HIF1AN) in Glioblastoma Multiforme (GBM).** The data has been extracted from ONCOMINE database, which compared FIH-1 mRNA levels in normal brain tissues from Epilepsy patients (Class I) and in tumor tissues from GBM patients (Class II). There is a significant reduction of FIH-1 mRNA levels in tumor tissues compared to normal brain tissues.
(TIF)
