# Supplementary figures and images for: The Role of Factor Inhibiting HIF (FIH-1) in Inhibiting HIF-1 Transcriptional Activity in Glioblastoma Multiforme
Source: PLoS One. 2014 Jan 23;9(1):e86102. doi: 10.1371/journal.pone.0086102 (PMC3900478; doi:10.1371/journal.pone.0086102)

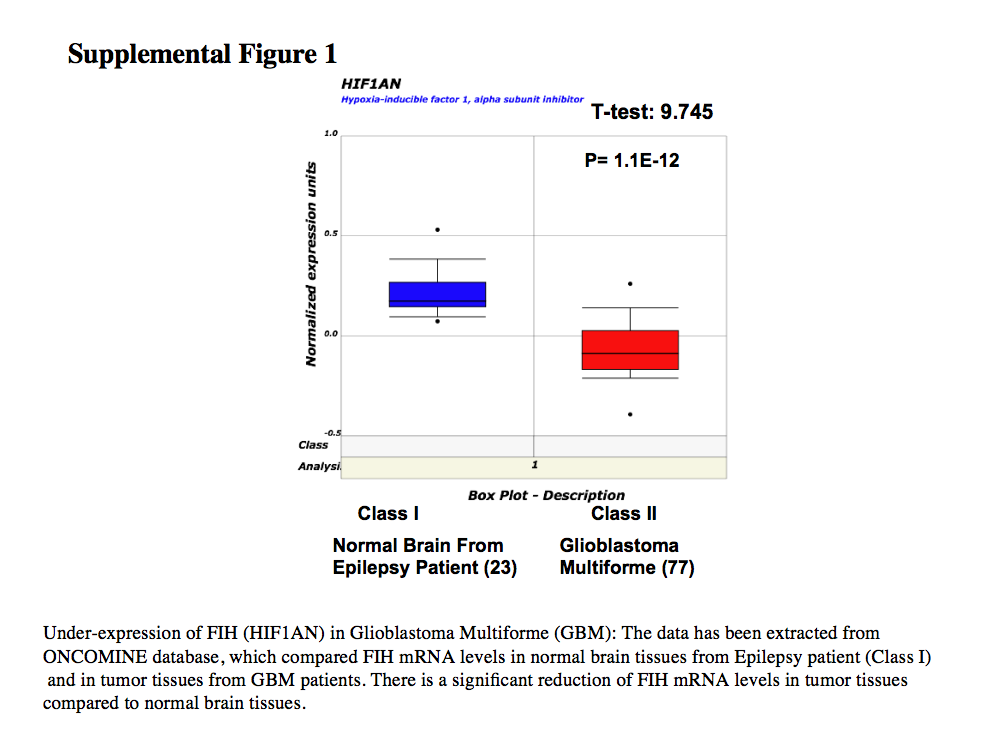

Supplement: Figure S1 — Under-expression of FIH-1 (HIF1AN) in Glioblastoma Multiforme (GBM). The data has been extracted from ONCOMINE database, which compared FIH-1 mRNA levels in normal brain tissues from Epilepsy patients (Class I) and in tumor tissues from GBM patients (Class II). There is a significant reduction of FIH-1 mRNA levels in tumor tissues compared to normal brain tissues. (TIF) [file pone.0086102.s001.tif]
